# Repeatability and Reproducibility of Macular Hole Size Measurements Using Optical Coherence Tomography

**DOI:** 10.3390/jcm10132899

**Published:** 2021-06-29

**Authors:** Carmen Baumann, Ahmed Almarzooqi, Katharina Blobner, Daniel Zapp, Katharina Kirchmair, Lydia S. Schwer, Chris P. Lohmann, Stephen B. Kaye

**Affiliations:** 1 Ophthalmology Department, Technical University of Munich (TUM), 81675 Munich, Germany; c.baumann.org@gmail.com (C.B.); draamarzouqi@gmail.com (A.A.); Katharina.Blobner@mri.tum.de (K.B.); Daniel.Zapp@mri.tum.de (D.Z.); Katharina.Kirchmair@mri.tum.de (K.K.); LydiaStella.Schwer@mri.tum.de (L.S.S.); Chris.Lohmann@mri.tum.de (C.P.L.); 2 Department of Eye and Vision Science, University of Liverpool, William Henry Duncan Building, 6 West Derby Street, Liverpool L7 8TX, UK

**Keywords:** macular hole, optical coherence tomography, scan pattern, minimum linear diameter

## Abstract

The purpose of this study was to assess the repeatability and reproducibility of measuring the minimum linear diameter (MLD) of macular holes (MHs) using horizontal linear and radial scan modes in optical coherence tomography (OCT). Patients with concurrent sets of radial and horizontal linear OCT volume scans were included. The MLD was measured twice in both scan modes by six raters of three different experience levels (groups). Outcome measures were the reliability and repeatability of MLD measurements. Fifty patients were included. Mean MLD was 317.21(±170.63) µm in horizontal linear and 364.52 (±161.71) µm in radial mode, a difference of 47.31 (±26.48) µm (*p* < 0.001). In the radial scan mode, MLD was identified within 15° of the horizontal meridian in 27% and within 15° of the vertical meridian in 26.7%, with the remainder (46.3%) in oblique meridians. The intra-group coefficients of repeatability (CR) for horizontal linear mode were 23 µm, 33 µm and 45 µm, and for radial mode 25 µm, 44 µm and 57 µm for groups 1, 2 and 3, respectively. The inter-group CR, taking group 1 as reference standard for groups 2 and 3, were 74 µm and 71 µm for the linear mode, and 62 µm and 78 µm for radial mode. The radial mode provides good repeatability and reliability for measurement of MLD. In a majority of cases the MLD does not lie in the horizontal meridian and would be underestimated using a horizontal OCT mode.

## 1. Introduction

Optical coherence tomography (OCT) is the standard diagnostic tool for analysing the configuration and the minimum linear diameter (MLD) size of a macular hole (MH), which is used as a guide to the management of MHs. It is apparent, therefore, that accurate and precise measurement of the MLD is important.

For the measurement of a MH, contemporary OCT devices offer two different macular scanning profiles; one is a raster of horizontal line scans and the other one consists of radial scans around a central point. In both modes, the scan is selected which displays the widest dimension of the MH and the MLD is then measured manually using the built-in softer calliper tool. The horizontal linear raster scan is currently the routine mode that is most used in clinical settings as well as in major large MH trials [1,2,3,4,5,6] and has formed the basis for clinical guidelines.

It is known, however, that the shape of a MH is not uniform [7] and the widest aperture may lie in any meridian. Using the horizontal mode, therefore, will not enable measurement of the MLD when it lies in an oblique meridian MH. Furthermore, radial scans have already been shown to provide a higher detection rate of small MHs compared to horizontal linear scans [8]. The intra- and inter-observer agreement, however, of identifying the section containing the MLD of a MH in the radial mode is unclear and needs to be assessed before wider routine use of the radial mode is translated into clinical practice. The purpose of this study, therefore, was to compare the reliability and repeatability of MH measurements in horizontal and radial OCT modes.

## 2. Materials and Methods

Patients with MHs who had sets of concurrent radial and horizontal linear volume OCT scans of primary MHs (obtained in the same examination session) were included. Scans were randomly extracted from a large OCT database (Heidelberg Spectralis, Heidelberg Engineering, Heidelberg, Germany, standard 6 mm macular scans with 19 raster lines and radial volume scans with 24 sections) at the Ophthalmology Department, Technical University of Munich, Germany. Since fixation is impaired in eyes with a MH, patients were presented with a fixation target light to their contralateral eye to obtain steady fixation while the OCTs were taken, with manual centration of the OCT raster on the centre of the MH in both modes.

Inclusion criteria were the availability of both a linear and a radial volume scan taken concurrently with good quality images of primary full thickness MHs of all sizes. Exclusion criteria were scans not centred on the MH, impending and lamellar MH and co-existing macular pathology.

Six raters participated: four vitreoretinal consultants (raters 1 to 4) and two third year ophthalmology specialist trainees (raters 5 and 6). Raters 1 and 2 were involved in research on MHs and were therefore assessing the largest number of MHs on a routine base. To analyse the influence of experience in MH measurements, the raters were sub-grouped into group 1 (raters 1 and 2), group 2 (raters 3 and 4) and group 3 (raters 5 and 6).

The International Vitreomacular Traction Study Group Classification System method [1] was used to measure the size of the MHs. All raters received instructions and interactive example demonstrations on how the MLD should be measured using the Heidelberg Eye Explorer viewing module. Using the 1:1 µm mode and starting in the radial volume scan mode, the raters estimated and selected the meridian of the scan containing the largest dimension of the MH. In this scan, the narrowest distance between the two edges of the MH, parallel to the RPE, was then measured (avoiding the area of the operculum if present) using the built-in software calliper of the device to determine the MLD. The raters then repeated this process in the horizontal linear raster volume scan mode. Four weeks later the raters repeated the task on the same MHs, this time commencing in the horizontal linear scan mode. The raters were masked to their previous as well as to the other raters’ measurements. In order to determine the shape of the MHs at the level of the MLD, the section of the MH at 90° to the MLD was also measured, and the ratio between these two measures, with the MH MLD size in the denominator, was calculated. The closer this ratio (index of the ovality of the MH at the level of the MLD) to unity, the more round the MH at the level of the MLD.

### Statistical Analysis

The data were collected and analysed using SPSS V.27.0 (SPSS inc, Chicago, IL, USA). A Shapiro–Wilk’s test was used to assess normality of the distributions. Continuous variables are reported as mean (±SD). For the repeat measurements of each rater the 95% confidence interval (CI) and the upper and lower limits of agreement (LOA) were determined. The difference between the two measurements for each MH was plotted against their mean for both the horizontal linear and the radial OCT modes (Bland–Altman plots) [9]. This was done first for each rater and then for each group of raters. Coefficient of repeatability (CR) was used to determine intra-individual repeatability with CR = 2 × SD of the repeated sets of measurements [10]. A Levene’s test was performed to assess the homogeneity of variance within the two repeat sets of measurements of each rater, in both the linear and the radial modes. An F test was used to test the variance of the differences within and between raters and the groups. An analysis of variance (ANOVA) was performed to test for proportional bias and to compare means between groups. A general linear multivariable model was used with MLD size as the dependent variable and OCT mode, experience and time of rating as covariates and factors. Significance was *p* < 0.05 and a Sidak correction was used for multiple tests.

## 3. Results

Mean MLD in the linear scan mode was 317.21 (±170.63) µm (range 65 µm to 803 µm) and 364.52 (±161.71) µm (range 109 µm to 806 µm) in the radial scan mode (*p* < 0.001). The mean difference between the MLD in radial and the horizontal linear mode was 45.99 (±59.73) µm in group 1, 50.28 (±59.31) µm in group 2 and 45.67 (±64.40) µm in group 3, with a mean difference of 47.31 µm (±26.48) (*p* < 0.001).

**Figure 1:**
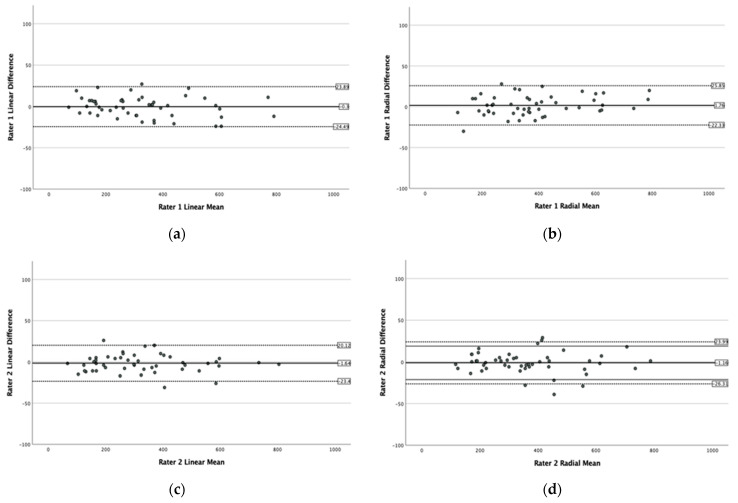

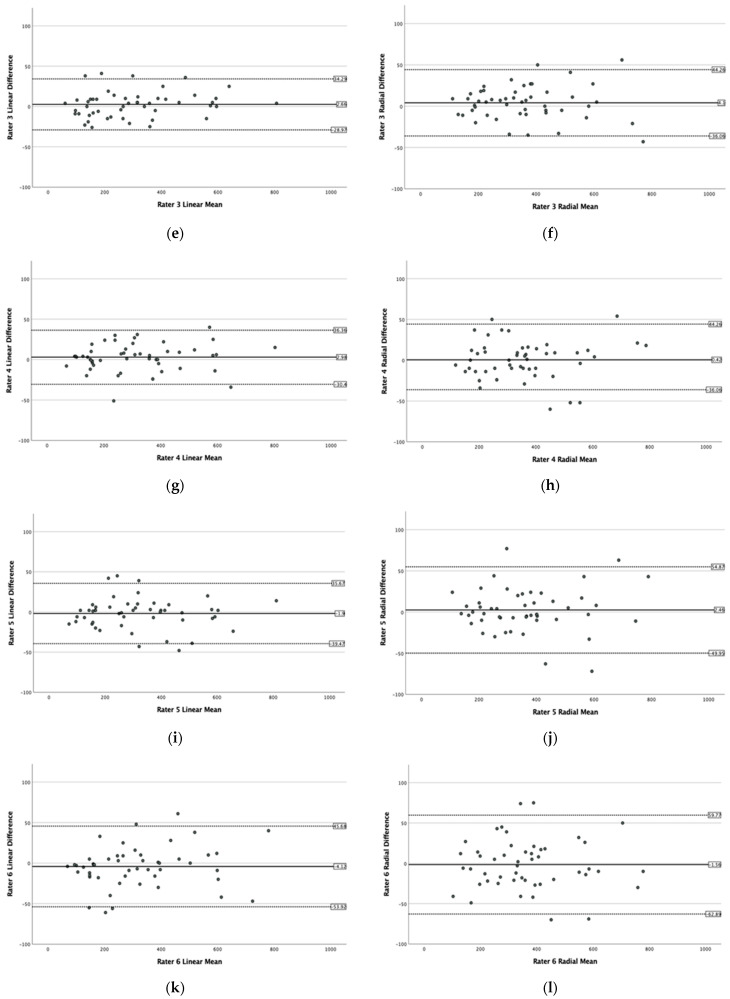
Bland–Altman plots of the MH size (MLD) measurements obtained by the six raters in the horizontal linear and the radial OCT scan modes with no systematic bias. (**a**–**l**) The *x*-axis displays the mean size of the MLD measurements of the MHs and the *y*-axis, the difference between the two readings of each rater.

**Figure 2:**
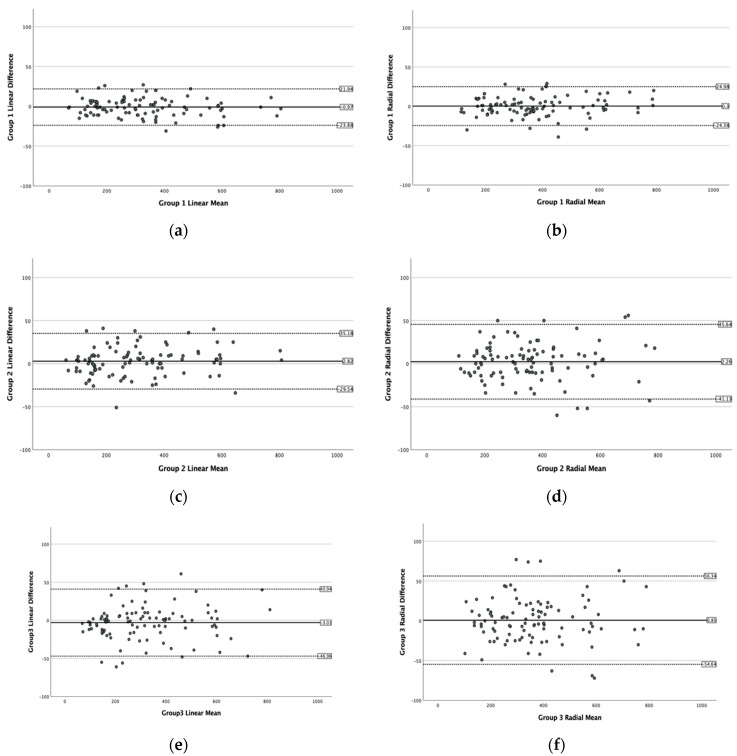
Bland–Altman plots of the MH size (MLD) measurements obtained by the three groups in the horizontal linear and the radial OCT scan modes with no sign of systematic bias. (**a**–**f**) The *x*-axis displays the mean size of the MLD measurements of the MHs and the *y*-axis the differences between the readings within each group.

For both the linear and radial OCT mode measurements, homogeneity of variance of the differences in MLD measurements across all MH sizes was confirmed with a Levene’s test between raters (*p* = 0.999 and *p* = 0.995, respectively) as well as between groups (*p* = 0.952 and *p* = 0.867, respectively), demonstrating that the differences of the measurements were not related to the mean and there was no significant increase in the variability of the differences as the magnitude of the measurements increased. None of the twelve Bland–Altman plots for the individual raters (Figure 1) nor the six Bland–Altman plots for the three groups of raters (Figure 2) demonstrated any systematic bias for the repeat measurements.

There was no association between the MLD measurements and experience level, either in general (*p* = 0.74) or within the horizontal (*p* = 0.71) and radial modes (*p* = 0.79). There was, however, a significant association of MLD measurements and OCT mode (*p* < 0.001) but not with the experience of the rater (*p* = 0.84) or over the period between measurements (test vs. re-test, *p* = 0.66).

### 3.1. Repeatability and Reproducibility

For both the horizontal and radial modes, there were no significant differences in MLD measurements, either within raters (*p* ≥ 0.22 and *p* ≥ 0.16) or groups (*p* ≥ 0.91 and *p* ≥ 0.31), or between raters (*p* = 0.98 and *p* = 0.97) and groups (*p* = 0.76 and *p* = 0.81).

For both the linear and the radial modes, however, there was a significant increase in variability from group 1 to group 2 (*p* < 0.01, *p* < 0.01), group 1 to group 3 (*p* < 0.01, *p* < 0.01) and from group 2 to group 3 (*p* = 0.015, *p* = 0.003). The intra- and inter-observer and group intraclass correlation coefficients (ICC) within and between the six raters and the three groups were all ≥0.98. As proposed by Bland and Altman, the 95% limits of agreement (LOA) contain 95% of the differences between repeated measurements on the same subject and two repeat measurements of the same subject will be within 1.96 × SD of the differences for 95% of subjects, which represents the coefficient of repeatability (CR).

To assess the repeatability of measurements for raters 1, 2, 3, 4, 5 and 6, the intra-rater coefficients of repeatability (CR = 2 × SD) were calculated, and to evaluate the reliability of measurements between the raters, the 95% LOAs for the inter-rater repeatability were calculated using the primary measurements of each rater and defining rater 1 as the nominal reference standard. To analyse the repeatability of measurements for groups 1, 2 and 3, the intra-group CR was determined and to investigate the reproducibility of measurements between the groups, the inter-group CR was calculated, taking group 1 as nominal reference standard for group 2 and group 3. This was performed for both the horizontal linear and the radial OCT scan modes (Table 1).

### 3.2. Meridian of MLD Selection in the Radial OCT Mode

**Figure 3:**
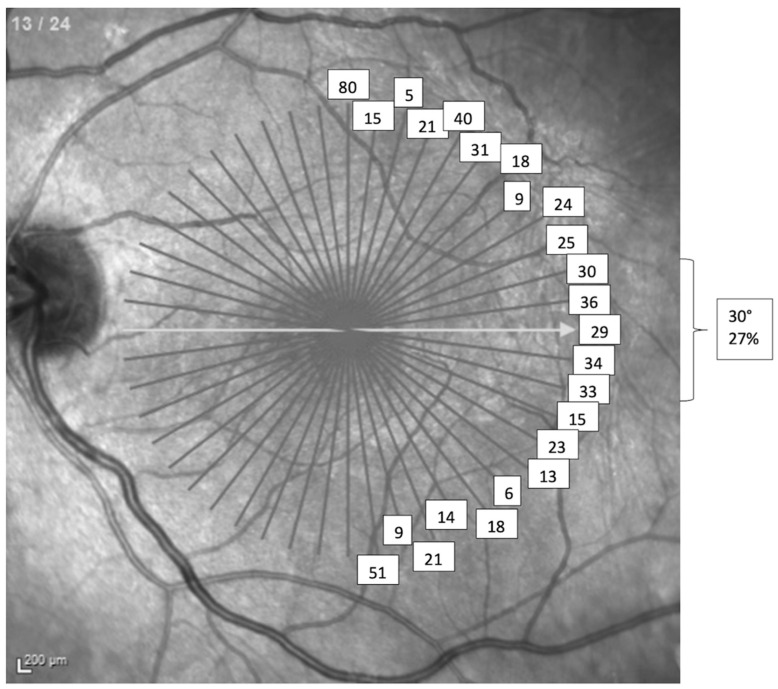
Distribution of the MLD orientation of the 50 MH, measured by the six raters in the test and re-test (*n* = 600 readings). The sections selected for MLD size measurements varied 360° with no predilection of the horizontal meridian, therefore requiring a radial scan pattern to detect the MLD.

The distribution of the MLD orientation of the 50 MH, measured by the six raters in the test and re-test (600 readings in total), are shown in Figure 3. The MLD was selected within 15° of the horizontal meridian in 27% of cases, within 15° of the vertical meridian in 26.7% of cases and in a diagonal meridian (greater than 15° from the horizontal or vertical meridians) in 46.3% of cases.

The distance in degrees between the two sections each rater chose to measure the MLD in the test and re-test of the same MH gave a mean distance between the corresponding sections of 8.0° in group 1, 15.3° in group 2 and 15.0° in group 3. The mean ratio between the MLD of the MHs and the MLD in the section at 90 degrees to the former was 0.68 (±0.21). As this ratio increased (i.e., the MH at the level of the MLD was more round) the variation between the selection of a certain section (meridian) to measure the MLD by each rater and group also increased (R^2^ = 0.33, *p* < 0.01).

## 4. Discussion

We found that there were no significant differences in the mean MLD measurements, neither within nor between raters and groups, with very high ICCs. Antonopoulou et al., compared the intra- and inter-individual repeatability in MLD between three consultant vitreoretinal surgeons and three fellows using horizontal line scans and also found no significant difference in the intra-individual repeatability, their CR (43 µm to 73 µm) being slightly higher than ours (22 µm to 51 µm) [11]. Their 95% LOA for inter-observer agreement demonstrated a larger variance of CR (62 to 122 µm) compared to our results for both the horizontal (59 to 70 µm) and radial (59 to 73 µm) modes. We did, however, find the variation between repeated measurements to be significantly higher in less experienced raters, which would suggest that a level of training is required to obtain more repeatable MLD readings.

In our study the mean MLD size recorded in the horizontal linear scan mode (317 µm) underestimated the size of the MHs when obtained in the radial mode (365 µm) by 13%. This difference was independent of the experience level of the rater. Using the definition of small MHs having a MLD of ≤250 µm and large MHs of >400 µm, we found that reliance on the horizontal linear as opposed to the radial OCT scan mode resulted in a different classification of 11/50 MH (22%). While horizontal line scans serve well in displaying macular pathology affecting larger areas of the macula (e.g., in age-related macular degeneration or diabetic macular edema), it may not be the optimum mode for measuring MHs, which are confined to the centre of the macula. Using horizontal linear scan patterns relies on the MHs being symmetrical in all dimensions, i.e., a circle. Our findings support that of Chen et al. [7], that MHs are irregular in shape, with differing diameters across the 360° defect. In our series the mean ovality ratio was 0.68, and with the largest MH diameter potentially found in any meridian. In addition, only 27% of MHs had their MLD within 15° of the horizontal meridian. This indicates that 73% of MH had their MLD >15° away from the horizontal plane and would account for the underestimation of the MLD if assessed in the horizontal linear mode. For the same reason radial scans have already been shown to provide a higher detection rate of small MH as compared to horizontal line scans [8]. The more oval a MH, the easier it is to select the section with the largest MLD. We used the ratio of the MLD at 90° to the section of the MH MLD measurement (maximum MLD) to reflect the overall shape of the MH. We found that the smaller the ratio (the more oval shaped or less round MH are), the closer the agreement amongst raters in selecting the same radial section. Conversely, the higher the ratio (the more round the MH) the less agreement exists between raters in the selection of the radial section, and the actual difference in MLD at 90° was small as a reflection of the similarity of the MLD in all meridians.

While the MLD is used to define the size of MHs in most large MH trials [1,2,3,4,5,6], other MH characteristics such as the base diameter have also been shown to correlate with anatomical and functional outcomes after MH repair [12]. Furthermore, in clinical practise it may be difficult and time consuming to always accurately choose the meridian with the OCT scan section that displays the widest MLD of a MH, and the readings are subject to observer error. Therefore, development of software applications that would perform automated detection of the relevant meridian and automatically calculate the largest MLD is wanted.

The current study has several limitations. The scan protocols used were standard protocols used in clinical settings and not high-density rasters. It should be noted, however, that the minimum requirement by The International Vitreomacular Traction Study Group Classification System method [1], which has formed the base for many studies on MHs, is a single centred horizontal line across the MH to obtain MLD measurements. High-density volume scans would more reliably detect the true MLD of a MH, but even a high-density horizontal raster scan can only detect the MLD of a MH if it lies in the horizontal meridian. Otherwise, it would only be truly reflected by a 360-degree radial scan profile.

A criticism of the radial mode is that it can be difficult to locate the scan on the centre of the MH and decentration may reduce measurement accuracy and precision, leading to overestimation of the ovality of the MH, hence experienced technicians are required.

Despite these limitations, we have shown that the use of the radial mode provides comparably good repeatability and reliability to the horizontal mode for MLD measurement of MHs. In a majority of cases the MLD does not lie in the horizontal meridian and would be underestimated using a horizontal OCT mode.

## 5. Conclusions

The radial OCT scan mode provides good repeatability and reliability for measurement of MLD. In a majority of cases the MLD does not lie in the horizontal meridian and would be underestimated in the horizontal linear mode.

## Figures and Tables

**Table 1:** Coefficients of repeatability CR (2SD) for the repeatability and reproducibility of measurements between and within raters and groups in the horizontal linear and the radial OCT scan modes.

CR	OCT Mode	Rater 1	Rater 2	Rater 3	Rater 4	Rater 5	Rater 6
Intra-rater	Linear	25	22	32	34	38	51
Intra-rater	Radial	25	26	41	48	53	63
Inter-rater	Linear	Reference	62	70	60	59	64
Inter-rater	Radial	Reference	59	61	64	63	73
		Group 1		Group 2		Group 3	
Intra-group	Linear	23		33		45	
Intra-group	Radial	25		44		57	
Inter-group	Linear	Reference		74		71	
Inter-group	Radial	Reference		62		78	

## Data Availability

Not applicable.
